# Collectanea Medica: Consisting of Anecdotes, Facts, Extracts, Illustrations, &c.

**Published:** 1826-03

**Authors:** 


### COLLECTANEA MEDICA:

CONSISTING OF

### ANECDOTES, FACTS, EXTRACTS, ILLUSTRATIONS, &c.

*Relating to the History or the Art of Medicine, and the Collateral Sciences.*

Floriferus, ut apes, in saltibus omnia libant,
Omnia nos, itidem, depascimur aurea dicta.

Art. I.—*Extracts from "A Treatise on the Diseases of the Eye; including the Doctrines and Practice of the most eminent Modern Surgeons, and particularly those of* Professor Beer. By George Frick, m.d. Ophthalmic Surgeon to the Baltimore General Dispensary. *A new Edition, with Notes,* by Richard Welbank, Member of the Royal College of Surgeons, and of the Medical and Chirurgical Society of London."—Anderson, London, 1826.

Part I. Chap. III.—*Rheumatic Ophthalmia,* (*Ophthalmia Rheumatica. Sclerotitis.*)

Some authors, and among the rest Professor Walther, have termed this species of ophthalmia the *arthritica.* The epithet *rheumatica,* given it by Beer, is perhaps the most appropriate; not only because the texture in which it is seated is analogous to that in which rheumatism is commonly found to have its seat, viz. the ligamentous and fibrous parts of the body, but because it is most generally known to alternate with, or succeed to, rheumatic affections in other parts of the system, and is most prevalent in those seasons of the year when rheumatism is most frequent.

The disease generally commences with a deep, lancinating, or acute pain of the orbit, now and then confined to the eyebrow, but most commonly extending over the whole of the side of the head which is affected.* This pain is rendered worse at night, when the patient is covered warmly in bed; and very often one or more of the joints are affected at the same time. Sometimes general rheumatism, or pains in the limbs, have preceded the inflammation in the eye, which are mitigated, or cease entirely, as soon as the disease is once firmly established in the latter organ. With the first sensation of pain in the eye, there will be found more or less redness of the sclerotica, epiphora, and intolerance of light. The redness is to be distinguished from that which takes place when the conjunctiva is the seat of inflammation, from its being of a lively carmine or rose colour, from its being diffused over the whole sclerotica, and from its being more deeply situated.† There is a constant weeping of the eye, more particularly when it is subjected to sudden vicissitudes of temperature, or exposed to a damp and cold atmosphere. Hence this variety of ophthalmia has been termed by the older nosologists, the *ophthalmia humida.*

These symptoms, if not controlled by art, are every day increased. The redness of the eye becomes greater, extending itself to the vessels of the conjunctiva, which may be seen of a florid red colour, and covering the more delicate rose-like capillaries of the sclerotica. The pain is rendered more severe, affecting not only the cranium, but the whole head and face.‡

The cornea, or rather the conjunctiva covering this tunic, participates in the inflammation. It is observed to lose its transparency, and assume a dull horny appearance. Small vesicles, or phlyctenulæ, form on various parts of this membrane; which bursting, pour forth their contents, and leave behind as many superficial ulcers, which seldom or never, however, extend deep into the cornea. If the constitution be otherwise healthy, they most commonly disappear with the other symptoms of redness, pain, &c. leaving in general little or no opacity, or any further trace of their having once existed, than some slight and almost imperceptible excavations or foveæ of the cornea; which, however, disappear altogether in the course of a very little time.

When the disease is permitted to run its course, the inflammation spreads to the iris,—the vessels of the sclerotica are seen to take on the beautiful rose-coloured zone peculiar to iritis, the iris loses its irritabi-

* In many of these cases, before the inflammatory action is established, I have derived the most gratifying advantage from the exhibition of ʒss. of muriate of ammonia, or ℈ij. of the subcarbonate of iron, every four hours, in a glass of water.—Editor.

† There is no pale line of interval between the edge of the cornea and the zone of sclerotic vessels, where the sclerotitis is acute.—Ed.

‡ Perhaps some forms of remittent ophthalmy are attached to the class now under consideration. An interesting practical case, attended with intense suffering, and relieved by large doses of opium, is related by Dr. Curry, who was himself the subject of it, in the Medical and Chirurgical Transactions, vol. iii. p. 348. —Editor.

lity and becomes sluggish; its colour is changed to a greenish hue. The pupil is irregular, angular, and contracted. There is effusion of coagulable lymph in the pupil, which considerably impairs the patient's vision; and small brownish condylomata are seen to arise at the same time upon the iris.

Sometimes the inflammation, instead of attacking the anterior hemisphere of the eye, is propagated to the more interior tunics, the choroid, retina, and hyaloid membranes. If the eye be now examined, a dark greenish opacity, of a concave appearance, will be distinctly observable deep within the pupil, becoming daily more apparent, until it extends quite to the pupil. The vision is more or less impaired, or even entirely destroyed. If the disease be not arrested at this period, the vitreous humour enlarges, so that the volumen of the eye is considerably increased; and we have superadded to the opacity of the vitreous humour, or *glaucoma*, hydropthalmia.

The causes which produce inflammation of the sclerotica are, in general, the same as those which excite inflammation in other organs of the body. Most commonly the disease may be traced to sudden exposure to cold, after the body has been preternaturally heated. It is often preceded by pains in the limbs, or some general rheumatic affection: more frequently the pain and redness of the eye are the first symptoms of the disease, or it is ushered in by chill and fever. The disease generally makes its attack in one eye only, and the other is not affected until the inflammation has made some progress, or entirely exhausted itself in the first. At other times both eyes are attacked simultaneously, and the disease advances equally in both.

*Cure.*—When the disease is violent, accompanied with a tense and hard pulse, it will be necessary, in the first stage, to have recourse to venesection. This may be aided by cupping, or leeches applied to the temples. The division or opening of the temporal artery, as uniting the advantages of local with general depletion, has been highly recommended in this species of ophthalmia; but is now very generally abandoned, as it is constantly followed by an aggravation of the disease. Dr. Vetch has very satisfactorily accounted for this, in the power which arteries possess of rapidly accommodating themselves to the exigency of the part which they supply.

As this ophthalmia is, in general, accompanied with some affection of the stomach or alimentary canal, it is advisable, in most cases, to follow the bleeding by an emetic, or to empty the bowels by some active cathartic. To these should succeed the employment of antimonials, given in small quantities, so as to keep up a constant nausea, and determine towards the skin. They are more especially advantageous where the disease has originated from exposure to cold, or where the functions of the skin have been suddenly suppressed.*

* Mr. Brodie suggests the use of colchicum, as advantageous in the cases which attend, or succeed to, puriform inflammation of the urethra, with rheumatism of the joints. Sometimes the state of constitution is brought on by drinking light wines, cider, or hard beer. The presence of acidity in the stomach is always worthy attention in gouty and rheumatic subjects. Decoction of sarsaparilla in lime-water is a valuable medicine in some of these cases.—Editor.

Mr. Wardrop has used, with considerable advantage, small doses of bark in rheumatic ophthalmia, and considers it to possess as specific an effect in this disease as in ague. The practice, from the greater violence of inflammatory affections in this country, is scarcely admissible.

The pain in this disease is best alleviated by blisters applied behind the ears, or to the nape of the neck; warm fomentations of poppy-heads, hyosciamus, &c. contribute to the same effect. Where the pain is extremely violent, and accompanied with a sensation of tension or constriction of the globe, relief is frequently obtained by evacuating the aqueous humour, in the manner proposed by Mr. Wardrop.

Unctuous or fluid applications to the eye are seldom of much benefit, excepting in the latter state of the inflammation. The most useful of these are the vinous tincture of opium, or Sydenham's tincture, streaked once or twice daily, by means of a delicate camel-hair pencil, into the eye.

### PART II. CHAP. III.—*Of the Diseases of the Iris.*

*Mydriasis.*—This name is applied to the extraordinary and permanent dilatation of the pupil. The transient dilatation which is caused when the eye is withdrawn from a lighter into a darker region, is dependent upon the peculiar organisation of the iris, and cannot, therefore, fall under our consideration at present.

This affection most commonly attacks both eyes at the same time: often, however, the pupil of one eye only is dilated, whilst the other preserves its natural size. The form of the pupil is commonly the same as in the healthy state; at other times it is oval, elongated, or angular.

Mydriasis may be congenital or acquired, symptomatic or idiopathic. That species of mydriasis which is congenital, and consequently incurable, is most commonly symptomatic, depending upon a want of sensibility in the optic nerve, or its expansion into the retina. These cases generally terminate in complete gutta serena.

Long confinement in a dark room may render habitual that dilatation of the pupil, which the eye naturally suffers on being deprived of its proper stimulus, light. It would appear that, like all other organs of the body, the iris, from want of proper exercise, loses its power of action. Persons who have passed a number of years in dark dungeons and gloomy cells, afford remarkable instances of the influence of habit on the motions of the iris.

Mydriasis is often a symptom only of some other disease, as amaurosis, worms in the intestines, organic affections of the brain, as apoplexy, hysteria, epilepsy, hydrocephalus, &c. The pupil is always found dilated during sleep, as well as after death. Persons affected with hydrophthalmia have the pupil much dilated, in consequence of the great development of the vitreous humour. Hence, too, we find mydriasis often a symptom of cataract, more especially of the soft or milky kind, where the lens is much enlarged.

Idiopathic cases of mydriasis are much more rare than the sympathetic. They are most commonly induced by blows upon the eye injuring the frontal eye; an effect which may probably be accounted for, from the connexion of the frontal branch of the fifth pair of nerves with those supplying the iris. Sometimes they are caused by the too sudden and forcible expulsion of the crystalline, in the extraction of cataract. The iris, being compressed or extended by the passage of this body through it, causes a relaxation or palsy of its tissue. Injury or violence done to the ciliary nerves and body, may cause a similar palsy of this membrane, and consequent dilatation of the pupil.

The diagnosis in mydriasis is readily made out. It is necessary merely to distinguish the symptomatic from the idiopathic species. In the former, our attention should be directed to the disease of which this dilatation is but the symptom; and, by relieving the one, we cure the other. In the idiopathic species, warm, stimulating, aromatic, and spirituous substances have been successively tried; as also scarifications, leeches, blisters, setons in the neck, purgatives, emetics, &c. In some instances these remedies have succeeded; in general, however, they have proved fruitless, more particularly where the disease has been congenital.

The pupil is also liable to excessive contraction, a disease known under the name of *Myosis*, (*Phthisis Pupillæ.*) It is in some cases even completely obliterated, when it is termed *Synizesis*. These two diseases differ merely in degree, both being ascribable to the same causes.

Myosis may be symptomatic or idiopathic. The former is the case where, from an undue degree of sensibility in the retina, caused by, or subsequent to, inflammation of the eye, the pupil is found extraordinarily contracted. Nature, in this manner, provides against the injurious effects which might otherwise result from the continual irritation of so delicate a membrane. Inflammation of the brain is a common cause of myosis.

The most frequent of all the causes of this contraction of the pupil, is a violent ophthalmia, spreading to the iris; or it may be the effect of iritis itself. Hence it is a common sequela of the operation for cataract.

Myosis is often the consequence of wounds of the iris, or of the detachment of this membrane from the ciliary ligament, by violent contusion of the eye.

Myosis often comes on without any previous inflammation, and without any apparent affection of the eye. Gouty persons, and particularly such as have suffered the operation for cataract, are most subject to the disease; and it may appear a week, months, or even years, after the operation, without any evident cause, or any previous pain or inflammation of the organ.

Persons compelled by their profession to fix their eyes continually upon minute or glaring objects, are liable to have the pupil very contracted,—as watchmakers, silversmiths, miniature painters, engravers, &c. This arises *ex consuetudine*. The pupil, to guard the retina from the injurious consequences of the too vivid light, contracts; and this contraction, produced at first from habit, becomes at length so firmly established, that it is impossible afterwards to dilate the pupil.

There is yet a species of myosis which may be termed the myosis *spuria*, where the pupil is closed by some extraneous matter. This may be a clot of blood from extravasation in either of the chambers, pus filling up these cavities, or some part of the lens left behind after the extraction of cataract. This extraneous matter is frequently the coagulable lymph which is thrown out from the iris, under a state of inflammation. I have seen the whole pupil filled with such a boss of lymph, so that the vision was completely destroyed; yet the whole was absorbed again in a few days, under proper treatment.

*Synechia.*—The iris is sometimes found united to the cornea, at other times to the capsule of the lens; and this has given origin to the terms *synechia anterior* and *posterior*. The former may be congenital; but it is most generally the consequence of wounds penetrating the cornea, abscesses forming between the coats of this membrane, producing fistulous openings, or operations upon the eye. The adhesion formed in this way is seldom general, but exists only at the part wounded, or at the inferior portion of the cornea, where abscesses are most liable to be seated. It is very readily recognized; the united portion of the iris, and corresponding portion of the pupil, being commonly more prominent and immoveable, whilst the rest of the iris and pupil maintain their natural form and mobility. The deformity of the pupil is in proportion as the adhesion is formed nearer the circumference of the cornea, or to the degree of dilatation at the time of its formation.

*Synechia posterior* may succeed to the same causes, but is most commonly the effect of deep-seated inflammation of the eye, or of iritis. The adhesion in these cases is most generally total between the iris and capsule; and the pupil, though immoveable, maintains its natural figure and situation. Where the adhesion is only partial, the pupil is more or less angular and distorted.

In the first case, the disease is always incurable. Where it is partial only, and the uniting medium consists of a small portion of coagulating lymph, much benefit will be derived from the use of the belladonna or hyosciamus, in the manner advised when speaking of iritis, aided by the internal administration of small doses of mercury. The same means may prove effectual even after this lymph has become organised.

To prevent this adhesion in cases of wounds of the eye, it has been advised to expose this organ to alternate changes of light and darkness, so as to cause the iris to dilate and contract, and thus oppose its union with the contiguous membranes. Such a practice, however, must always prove prejudicial, as it never fails to increase the inflammation which inevitably follows any violence inflicted upon so delicate and susceptible an organ.

*Prolapsus of the Iris,* (*Prolapsus Iridis.*)—This affection is also termed *staphyloma iridis*, and consists of a tumor, formed by the iris, protruding through an unnatural opening of the cornea. The tumor which results from this protrusion is necessarily of the colour of the iris. Its size varies from that of a pin's head to a small pea; and hence the different names of *myocephalon*, *melon*, *hylon*, &c. The form is, in general, irregular; its surface is rarely smooth, as in staphyloma of the cornea, but more commonly unequal or angular; the tumor is soft, and, when recent, easily reducible. As the cornea is seldom pierced in more than one spot, the prolapsus is most usually single; where this membrane is wounded in several places, the staphyloma may be multiple.

The patient complains of a pain, similar to that produced by a pin thrust into the eye, or of a sensation of tightness or constriction of the eyeball, like to that caused by a ligature around the organ. The pupil is always distorted, and drawn from its natural situation towards the aperture where the protrusion takes place; it loses its circular, and assumes an oval or oblong, form. Hence the distinctness, as well as the sphere of vision, is considerably impaired. To these symptoms are often superadded an habitual epiphora and inflammation, rendering the light excessively painful. By degrees the tumor hardens and becomes indolent, or gradually disappears; so that, after a certain time, nothing more is perceptible than a small blackish point in the midst of the cicatrix. The pupil, however, remains in the state before described.

The diagnosis in this disease is easily made out: the appearance of a tumor after a wound or ulceration of the cornea, and a deformity of the pupil, are its pathognomonic signs.

Some surgeons have advised the prolapsed iris to be returned into its natural situation, by means of an ivory stylet, or the common curette. This, however, is in general impracticable. Where the prolapsed part is large, it should be snipped off with a pair of scissors; after which the wound may be slightly touched with the caustic pencil. In this manner the adhesive action is soon set up, and an union takes place between the iris and cornea. Where the prolapsus is of long standing, and does not yield to the caustic, it may be removed with the cornea knife or scissors.

### Part III. Chap. I.—*Of the Diseases of the Eyelids.*

Inflammation of the eyelids, like inflammation of the eyeball, has been subdivided by the German pathologists into several varieties, according as the disease is confined to one or other of the tissues which make up their structure, or is dependent upon general taint of the constitution. I shall notice the most important of these only.

*Of pure Inflammation of the Eyelids,* (*Blepharophthalmitis Idiopathica.*)—This disease most frequently originates in the upper eyelid, spreading thence to the lower, or extending itself backward upon the conjunctiva. It commences with a red, tense, and painful swelling of the borders, which gradually extends over the whole of the eyelid, and is attended with great heat and throbbing. The motions of the eyelid are much impaired, or entirely destroyed. The inflammation spreading to the lachrymal and meibomian glands, their secretions are suspended; the eyeball, in consequence, is rendered dry; and every attempt of the patient to move the lid causes excessive pain, or a sensation not unlike that produced by foreign bodies lodged beneath them. The nostril of the affected side, from the same cause, and from the shrivelled state or entire closure of the lachrymal puncta, being deprived of its natural moisture, obliges the patient to sneeze from the least irritation; and always accompanied with a considerable aggravation of the pain in the swelling, darting backwards into the orbit or head. This stage of the disease is generally attended with some degree of febrile disturbance.

If the inflammation be not soon dispersed, suppuration takes place. The redness now increases, becoming of a dark or purple hue; the swelling is more prominent, or assumes a conical shape; the pain is irregular, and of a burning or pulsatory kind. The tumor at length becomes softer in its centre, and is less sensible to the touch than before. The natural secretions from the lids are again restored, or poured forth more copiously than in health, so that the lids are firmly agglutinated during sleep. An uncommon feeling of cold and heaviness is experienced about the eye; and a fluctuation is now distinctly perceptible in the swelling, sufficiently characteristic of the presence of matter.

Where the disease occurs in sound constitutions, and is properly treated from the commencement, it may in most instances be resolved. If, on the other hand, the inflammation arises in a weakened constitution, or is injudiciously treated by the surgeon, it rapidly runs into the suppurative stage, or advances to gangrene. The mischief in such cases is not confined to the outer integuments alone, but the mortification may extend to the orbicularis muscle, and, when severe, attack the organ itself.

From the quantity of cellular substance contained in the eyelids, the abscess which forms after inflammation, is, in general, very extensive: hence the unpleasant train of evils which frequently results from suppuration of these parts. Among these may be accounted, 1st, a contraction or complete adhesion of the lachrymal canals, causing a permanent stillicidium; 2d, a prolapsus of the upper eyelid; 3d, inversion or eversion of the upper eyelid, and lagophthalmos from loss of substance; 4th, fistulous sinuses and caries about the bones of the orbit.

The cure of this disease, in its first stage, is easily accomplished by the use of cold and astringent lotions, together with leeches. The latter should never be applied directly to the eyelid, as they are apt to increase the swelling and congestion of the part. Much benefit, however, may be derived from their application behind the ears. Where there is much constitutional disturbance, general bleeding, with purgatives, should be premised, and the strictest antiphlogistic regimen enjoined upon the patient.

As soon as the disease shows any disposition to suppuration, or the swelling assumes the conical form already described, all thoughts of dissipating the tumor should be given up, and the suppuration assisted by mild emollient cataplasms. If the abscess be seated in the middle of the upper eyelid, it may, in general, be left to burst spontaneously; if near the canthi, or in the under eyelid, the matter should be let out with the lancet, as soon as the fluctuation is perceptible, and healed like the common abscess. When fistulous sinuses form about the part, or gangrene ensues, they should be treated by counter-openings, bark, and all such remedies as are usually employed for similar affections in other parts of the integuments.

*Erysipelatous Inflammation of the internal Canthus,* (*Anchylops Erysipelatosa Idiopathica.*)—This disease, which is frequently confounded with inflammation of the sac itself, possesses all the characteristic symptoms of erysipelas in other parts of the body. As long as the lachrymal sac continues unaffected, the swelling of the parts is equally diffused, and no particular hardness is discoverable in any portion of the tumor. More commonly, however, the inflammation extends to this organ and its ducts; and a hard, circumscribed, and very painful tumor is then distinctly felt just below the tendon of the orbicularis muscle. In some cases it possesses a darker or redder colour than the adjacent parts. The puncta are completely closed, so that there is a constant stillicidium of the tears over the cheeks: the nostril upon the side affected is, in consequence, rendered dry, and uncommonly sensible to the slightest irritations.

These appearances gradually subside, and give place to a new train of symptoms, or to the second stage of the complaint. The papillæ and ducts, if they have not suffered severely in the first stage, again resume their office, and the tears are absorbed and transmitted, as usual, to the sac ; the edges of the eyelids and lachrymal caruncle secrete a tough viscid mucus, so that the lids, during sleep, are firmly agglutinated. The lachrymal sac, if the inflammation has been severe, becomes filled with mucus, which is easily pressed out through the lachrymal ducts and puncta. An abscess now forms beneath the integuments, which opens either externally, or penetrates the fibres of the orbicularis muscle, and the anterior walls of the lachrymal sac. This latter circumstance is easily distinguished, not only by the mucus which is discharged in these cases with the matter, but from a quantity of tears which issue at the same time, unmixed indeed, with the rest of the contents. It is somewhat more difficult to distinguish the disease, before the abscess has burst, from mucocele, or a collection of mucus within the lachrymal sac; but, by a little attention to the history and progress of the symptoms, the diagnosis is easily made out. In the former, or abscess of the cellular substance, the tumor in its commencement is hard and elastic, and the fluctuation only evident at the decline of the disease: in mucocele, on the contrary, the fluctuation, if there be any present, is only perceptible at the commencement, the tumor becoming constantly firmer and more unyielding as the complaint advances.

The prognosis in this disease is generally favourable, more especially when the lachrymal sac continues free from inflammation ; and the only ill consequence resulting is a slight degree of stillicidium lachrymarum, which gradually disappears. The result, however, is less fortunate where the inflammation has attacked and destroyed the anterior walls of the sac, as a very obstinate and long-continued blennorhœa of the sac succeeds.

The treatment in the first stage of this complaint does not differ from that pursued in the erysipelatous inflammation of other parts of the body; and all that is necessary will be to foment the lids occasionally with cold water, to open the bowels by any mild laxative, and to administer afterwards small doses of the tartarised antimony. Unless the inflammation be very severe or extensive, it is seldom necessary to resort to general bleeding. Where suppuration is about to form, the cold applications are to be exchanged for warm poultices of bread and milk, and the abscess, when fully formed, should never be permitted to open spontaneously, lest the sac become involved in the affection; but, as soon as the slightest fluctuation is perceptible, we are to proceed to open the sac with the common lancet or bistouri. Should the abscess have burst before the surgeon is called to see the patient, he should avoid the introduction of all probes and syringes into the sac, as is too commonly practised, and content himself with washing out the abscess daily with a little tepid water, thrown in by means of a small syringe. The wound is afterwards to be dressed with a small tent of charpie, moistened with the vinous tincture of opium, care being taken not to push it so deep into the wound as to enter the lachrymal sac.

*Trichiasis, (Inversion of the Eyelid.) Distichiasis, (Double row of Eyelashes.)*—Trichiasis appears in one of two forms; either the cilia are turned inwards upon the eye, without any incurvation of the tarsus, or the tarsus itself is inverted together with the cilia. Some authors have given to the latter the name of *entropion*, to distinguish it from the former, or the trichiasis. It seldom happens that all the cilia of the same lid are inverted, unless there is at the same time a complete inversion of the tarsus; more commonly the disease is only partial, or confined to a small border of the lid. The effects produced by such inversion are the most distressing; each motion of the lid produces pain and irritation in the eyeball; and, from this incessant friction, there is kept up a continual weeping of the eye. In the ultimate stage of the disease, there is opacity of the cornea, with vessels overshooting its margin; or the most inveterate form of pannus is induced, and the patient is nearly or completely blind.

When, instead of a single, there exists a double row of cilia, inverted upon the eye, the disease is termed *Distichiasis.* Scarpa, and other writers of much distinction, have denied the existence of such a form of the disease. The fact, however, is proved by the very extensive experience and observations of Professor Beer. I have seen more than one instance where these pseudo-cilia were very apparent, and differed from the natural, not only in their position, but in their colour, form, length, &c. The disease very rarely attacks the whole of the lid, but appears in distinct and separate patches along its margin.

The causes of these diseases are not always very apparent. Most commonly they may be traced to inflammation of the inner lid or tarsus, which has terminated in ulceration and cicatrisation. The strumous ophthalmia of the glands which border upon the margin of the lids, is very apt to terminate in trichiasis. It was formerly a common termination of the variolous inflammation of these glands. It is a common sequela of psorophthalmy, if long neglected or improperly treated. The disease in some cases arises from a redundancy, or relaxation of the internal skin covering the palpebræ, from the loss of the natural elasticity of the tarsus, or from a thickened and callous fold of the conjunctiva lining the palpebræ.

*Cure.*—Where the disease depends upon the inversion of a few scattered cilia only, and is unconnected with any derangement in the structure of the tarsus or eyelids, it may, in general, be remedied by plucking out these hairs by means of a small forceps. The pseudo-cilia should be removed in the same manner.

When the disease is caused by an inversion of part or the whole of the tarsus, it is evident that the relief procured by pulling out the cilia can be but transient; and that the principal object of the surgeon should be to correct that morbid alteration of the cartilage which constitutes the disease. This is best and most effectually done by taking out a portion of the integuments opposite to that part of the cartilaginous border which is inverted. By the contraction and cicatrisation of this wound, the tarsus, together with the cilia, will be drawn outward into their natural position. The operation is best performed by the forceps of Bartisch, and the curved scissors. The surgeon, taking the instrument in his left hand, raises a fold of the skin immediately opposite the part where the trichiasis is greatest. Care should be taken, in raising up this fold, that no portion of the orbicularis muscle be included. The patient being directed to open his eye, the tarsus and cilia will be seen to have resumed their natural place and direction, provided a sufficient quantity of the integuments has been seized with the forceps. The fold, so included, is now to be cut off with a single stroke of the scissors. Little or no hemorrhage follows the operation, and this is easily checked by a little cold water.

If the excision be properly made, an oblong, oval-shaped wound will be the result. The lips of the wound are then brought together with sticking plaster, and a compress and bandage applied, as after the operation for cataract: it is seldom or never necessary to employ sutures.

Dr. Crampton's method of operation in cases of partial trichiasis, will be found to answer very effectually in removing the distortion. Conceiving the disease, in general, to originate from a thickened and contracted state of the conjunctiva, he has devised the following operation for its cure:—The eyelid being well turned out by an assistant, the surgeon, with his lancet, should divide the broad margin of the tarsus completely through, by two perpendicular incisions, one on each side of the inverted hair or hairs. This being done, the extremities of these perpendicular incisions should be united by a transverse section of the conjunctiva of the eyelid. The portion of cartilage contained within the incisions can then, if inverted, with ease be restored to its original situation, and retained there by small slips of adhesive plaster, or (perhaps what is better) by a suspensorium palpebræ, adapted to the length of the portion of the tarsus which it is intended to sustain.*

Mr. Saunders has recommended the excision of the tarsus itself, either in whole or in part, according to the extent of the disease;† but this is

---

* Vide *Essay on Entropion*, by R. Crampton.—London, 1815.

† The same operation was practised by Dr. Dorsey, of Philadelphia. Vide *Elements of Surgery*, by John S. Dorsey.—Philadelphia, 1818.

a most severe and tedious operation. Dr. Jæger, of Vienna, instead of removing the tarsus, takes away with a knife, or pair of scissors, the external border of the eyelid only, or that part which contains the cilia; and this practice has obtained the sanction of Professor Beer.

Where the disease is caused by a callous fold of the conjunctiva, it is easily removed by excising this part of the membrane.

### Chapter II.—*Of the Diseases of the Lachrymal Organs.*

*Inflammation of the Lachrymal Gland,* (*Dacryoadenitis.*)—The inflammation in this disease generally commences in the cellular substance which surrounds the gland; extending thence to the tissue which envelopes its acini, and seldom or never affecting the substance of the gland itself. The first symptoms which mark its invasion are an unpleasant dryness of the eye, and a fixed pulsatile pain of the temples or outer canthus, which extends into the orbit, or is propagated along the brows and temple to the jaws and ear. A hard, tense, and painful swelling is soon perceived in that part of the upper eyelid covering the lachrymal gland. The conjunctiva, excepting that portion of it near the external canthus, is seldom red or inflamed. From the gradual increase of the lachrymal gland, the eye is protruded from its socket, and the cornea is driven inward and downward towards the root of the nose. The motion of the globe outwards is generally impeded, from some affection of the rectus superior and externus muscles. Along with these symptoms, the sight is observed to decline more and more; the pupil contracts, or is immoveably fixed;* the pain increases, and there supervenes high inflammatory fever, accompanied with more or less of delirium.

If the inflammation is not discussed, the gland suppurates; the pain is now of a more throbbing kind, and the patient complains of an uneasy sensation of weight or coldness of the upper eyelid. A yellowish spot is observable on the conjunctiva of the bulb, or external surface of the eyelid, and a distinct fluctuation is felt in the tumor.

The disease is most common in young persons, and particularly such as are of a strumous habit. In the latter case, the inflammation is generally of a chronic nature, and the tumor may remain stationary for a long time. It is commonly excited by cold, or violent contusion of the gland; in some instances it has been caused by immoderate weeping, or by inflammation of the conjunctiva or eyelid.

As there is always danger lest the inflammation extend to other parts of the orbit, or involve the eye itself, it will be necessary, in the cure of this affection, to adhere to the strictest antiphlogistic measures. One or more general bleedings should be advised; after which we may resort to the use of leeches and blisters. These means may be assisted

---

* The affection of the iris, in diseases of the lachrymal gland, is easily accounted for, from the connexion of the nerves supplying this organ with the ciliary nerves, through the ophthalmic ganglion.

by cold lotions applied to the part, and the occasional use of purgatives.

If the inflammation do not yield to this treatment, or the swelling advances to suppuration, warm poultices of bread and milk, or fomentations of cicuta, hemlock, and the like, should be substituted for the cold lotions. The abscess should never be permitted to open spontaneously, as a troublesome fistulous sinus would prove the consequence. As soon, therefore, as the least fluctuation is perceptible in the tumor, the surgeon proceeds to open it with the lancet. The incision is seldom practicable from within; but is, in general, made from the external surface of the lid, and in a parallel direction with the fibres of the orbicularis muscle. The point of the lancet should be directed towards the groove of the gland, and the opening made of sufficient size to give free vent to the matter. The pain immediately subsides on the discharge of the matter, the protruded eyeball returns to its socket, and the vision gradually improves; but a greater or less degree of luscitas, or squinting, is generally the consequence. Sometimes the suppuration extends to the bones of the orbit, causing a troublesome fistula, and a continual discharge of an offensive ichorous fluid from the wound. The presence of caries may be easily ascertained by the introduction of a small probe.

More commonly, one or more of the excretory ducts of the gland are wounded, and there is a discharge of tears, or of tears mixed with matter, from the gland, *(fistula glandulæ lachrymalis;)* or a small capillary opening is left in the upper eyelid after the ulcer has already healed, through which there is a continual oozing of tears. The only mode of relieving such an affection is by the application of the lunar caustic, or by the actual cautery introduced to the bottom of the wound.

*Scirrhus of the Lachrymal Gland, (Scirrhus Glandulæ Lachrymalis.)*—The lachrymal gland, like the other glands of the body, may become the seat of scirrhous enlargement. The disease commences with a tumor near the external canthus, thrusting forward the superior palpebra. The eyeball is at the same time forced into the opposite direction, or downward and forwards, and in many instances is driven so far without the axis of vision as to impair considerably the sight. There is little or no pain in the commencement of the disease: as the gland enlarges, the patient experiences a sensation of pressure and tension, especially on moving the eye, and complains of an unpleasant dryness of the organ. If the tumor be examined, it will be found hard and unequal, or lobulated, and void of all fluctuation. The disease is at all times dangerous, as it seldom confines itself to the gland alone, but extends to all the adjacent parts.

Where the ordinary remedies for scirrhus prove unsuccessful, recourse should be had to extirpation. This should never be too long delayed, as the chance of success is always proportionate to its early performance. Professor Himley recommends the operation to be practised through the upper eyelid. It is preferable, perhaps, to divide the outer canthus, and dissect the tumor from beneath the lid. After the removal of the gland, the eye generally returns again to its former position in the orbit.*

*Hydatid of the Lachrymal Gland,* (*Hydatis Glandulæ Lachrymalis.*—This disease is of very rare occurrence. It commences with a dull pain about the orbit, which in a few days extends itself over one-half the head. The eyeball at the same time is rendered dry ; and, in a few weeks, from the great rapidity of the swelling, projects considerably out of the socket. If the eye be examined in the situation of the lachrymal gland, there will be found a tense elastic swelling, yielding slightly to any pressure made with the finger, but recovering its size again as soon as this pressure is removed. The progress of the disease is, in general, very rapid; so that, in a few weeks from the first attack, the patient complains of loss of rest, grows delirious, and eventually dies, with all the symptoms of confirmed apoplexy. The cornea, in the decline of the disease, assumes a dull glassy appearance. Under more favourable circumstances, the hydatid bursts, the parts suppurate, and a fistulous opening is formed, through which there is continually a small quantity of limpid fluid.

It is hardly possible to confound this disease with the scirrhous affection just described; the rapid progress and violence of the symptoms, and the distressing headache and delirium, are sufficiently characteristic of its nature. If more decisive evidence be wanting, it will be found in the spherical shape and peculiar elasticity of the tumor.

The disease may be in some measure relieved by puncturing the sac with a small lancet, and drawing off its contents. The puncture should be made in the most prominent part of the swelling, between the eyeball and lid ; and the sac, if possible, taken away with a pair of forceps. Where this is not practicable, the wound is to be kept open by a tent, as long as any fluid exudes, in order to destroy the sac.

---

* Vide Mr. Todd *on the Diseases of the Lachrymal Gland,* in the Dublin Hospital Reports, vol. iii.—Also Schmidt, *über die Krankheiten des Thränen-Organs.* Wien, 1803.

